# Volatile-Mediated Inhibitory Activity of Rhizobacteria as a Result of Multiple Factors Interaction: The Case of *Lysobacter capsici* AZ78

**DOI:** 10.3390/microorganisms8111761

**Published:** 2020-11-09

**Authors:** Anthi Vlassi, Andrea Nesler, Alexandra Parich, Gerardo Puopolo, Rainer Schuhmacher

**Affiliations:** 1 Department of Agrobiotechnology (IFA-Tulln), Institute of Bioanalytics and Agro-Metabolomics, University of Natural Resources and Life Sciences Vienna (BOKU), 3430 Tulln, Austria; anthi.vlassi@boku.ac.at (A.V.); alexandra.parich@boku.ac.at (A.P.); 2 Bi-PA nv (Biological Products for Agriculture), 1840 Londerzeel, Belgium; andrea.nesler@bi-pa.com; 3 Center of Agriculture, Food, Environment, University of Trento, 38098 San Michele all’Adige, Italy; 4 Research and Innovation Centre, Department of Sustainable Agro-Ecosystems and Bioresources, Fondazione Edmund Mach, 38098 San Michele all’Adige, Italy

**Keywords:** *Lysobacter capsici* AZ78, VOC, protein-rich medium, *Rhizoctonia solani*, ammonia, substrate pH, rhizobacteria

## Abstract

Plant beneficial rhizobacteria may antagonize soilborne plant pathogens by producing a vast array of volatile organic compounds (VOCs). The production of these compounds depends on the medium composition used for bacterial cell growth. Accordingly, *Lysobacter capsici* AZ78 (AZ78) grown on a protein-rich medium was previously found to emit volatile pyrazines with toxic activity against soilborne phypathogenic fungi and oomycetes. However, the discrepancy between the quantity of pyrazines in the gaseous phase and the minimum quantity needed to achieve inhibition of plant pathogens observed, lead us to further investigate the volatile-mediated inhibitory activity of AZ78. Here, we show that, besides VOCs, AZ78 cells produce ammonia in increased amounts when a protein-rich medium is used for bacterial growth. The production of this volatile compound caused the alkalinization of the physically separated culture medium where *Rhizoctonia solani* was inoculated subsequently. Results achieved in this work clearly demonstrate that VOC, ammonia and the growth medium alkalinization contribute to the overall inhibitory activity of AZ78 against *R. solani*. Thus, our findings suggest that the volatile-mediated inhibitory activity of rhizobacteria in protein-rich substrates can be regarded as a result of multiple factors interaction, rather than exclusively VOCs production.

## 1. Introduction

In the complex environment of the rhizosphere, plant–microbe and microbe–microbe interactions are partly facilitated by the release of various non-volatile and volatile compounds from all parties involved [1,2]. Bacteria inhabiting the rhizosphere, for instance, produce a plethora of secondary metabolites, which are involved in various processes such as intraspecies signaling, beneficial, as well as antagonistic interactions with other bacteria, fungi or plants. The latter is related to the production of soluble antibiotics and volatile compounds that can boost the defense of bacteria and assist them to compete for space and nutrients [3]. The production of volatile compounds by bacteria has been shown to be tightly linked to the composition of the growth medium used for cultivation [4,5,6,7]. Different types of volatile and non-volatile compounds were produced by bacteria when traditional synthetic growth media and media mimicking rhizosphere conditions were used, respectively [8,9], pointing to the importance of nutrient availability for the type of compounds synthesized.

Among the metabolites produced by bacteria to achieve antibiosis, volatile organic compounds (VOCs) have been increasingly studied in the recent years. Different types of bacterial VOCs have been shown to negatively affect the viability of various plant pathogenic fungi and oomycetes [10]. Pyrazines, for instance, are emitted by various rhizobacterial taxa and have been shown to contribute to their volatile-mediated inhibitory activity [6,11,12]. On the other hand, few reports exist on the contribution of other type of volatile compounds to the inhibition of plant pathogens, like inorganic ammonia that has been shown to be fungistatic [13] and is known to be produced by bacteria [14]. Ammonia is generally not covered during volatile analysis since this gaseous compound with low molecular weight and high polarity requires special gas chromatography (GC)-columns and instrumental conditions that are often not part of the commonly used gas chromatography–mass spectrometry (GC–MS) methods [15]. Previous studies on the composition of the volatile blend of bacteria have been focusing on the VOCs produced, utilizing common GC–MS methods for chemical analysis and thus possibly neglecting the contribution of ammonia to the bacterial volatile mixture. However, complementary to GC–MS methods, colorimetric assays have been successfully applied to detect ammonia in the bacterial volatile blend [16,17,18].

Soil can be enriched with ammonia by the emissions of microorganisms that either decompose dead organic matter rich in organic nitrogen (proteins, amino acids and nucleic acids) or degrade N-containing excretions of living organisms, such as urea [19]. Ammonia has been identified as one of the compounds associated with soil natural fungistasis [20,21,22]. Moreover, the addition of nitrogenous amendments in soil enhances natural decomposition, resulting in increased generation of ammonia that suppresses the growth of plant pathogenic fungi and oomycetes [23,24]. Soil bacteria (e.g., *Bacillus cereus*, *Enterobacter cloacae*, *Xanthomonas* sp., etc.) have been shown to emit ammonia [16,18,25,26,27,28,29] that in some cases suppressed the growth of their antagonistic plant pathogenic fungi and oomycetes [25,27]. In most of the cases, the use of substrates rich in proteins or amino acids was associated with the production of ammonia by bacteria in vitro [17,18,25,27], a phenomenon related to amino acid catabolism that involves, among others, deamination reactions which result in the subsequent release of ammonia gas [30].

Ammonia-mediated natural fungistasis has been associated with alkaline soils [31]. Indeed, soil pH is one of the most influential factors modulating the microbial community composition and dynamics [32]. In forest and arable soils with natural pH gradients, the fungal growth was favored by acidic conditions, while bacterial growth was favored by pH increase to more neutral or alkaline soil [32,33,34]. In vitro cultivation, most fungi prefer pH range of 5–6.5 for optimal growth, while most bacteria preferably grow on alkaline substrates with pH above 7 [35]. The bacteria-mediated inhibition of growth and sporulation of plant pathogenic fungi in vitro has been associated in some studies with pH changes of adjacent, physically separated substrates [4,36]. The change of pH in this case was considered more like an indicator of the bacterial volatiles’ basicity or acidity, rather than a factor contributing to the volatile compound-mediated inhibition [4,36].

*Lysobacter* sp. occupy a variety of ecological niches including freshwater and soil [37]. Species of the genus inhabiting soil have been associated with natural fungistasis [38] and a free living nitrogen-fixing strain that accumulates ammonia in vitro has additionally been characterized [39]. The genus constitutes a major source of lytic enzymes (e.g., chitinases), as well as antibacterial (e.g., WAP-8294A2) and antifungal (e.g., heat stable antifungal factor) secondary metabolites [40]. Furthermore, *Lysobacter* sp. have been characterized as producers of VOCs with anti-oomycete activity [6]. The biocontrol agent *L. capsici* AZ78 (AZ78) [41] can successfully suppress the growth of plant pathogenic oomycetes by implementing mycophagy [42], secretion of secondary metabolites [43] and lytic enzymes [44]. Recently, AZ78 was shown to be able to emit VOCs with inhibitory activity against plant pathogenic fungi and oomycetes [45]. However, the abundance of these metabolites in the blend of AZ78 volatiles could not fully explain the growth inhibition of plant pathogens in vitro [45]. In addition, former results on *Lysobacter* sp. VOCs showed a correlation between the type of volatiles produced and the type of growth media used [6]. Based on these observations, we tried in the present study to illustrate that further compounds and factors account for the volatile-mediated inhibitory activity of AZ78.

## 2. Materials and Methods

### 2.1. Bacteria and Fungi, Growth Media and Conditions

*L. capsici* AZ78, provided by Fondazione Edmund Mach, was routinely grown on Luria-Bertani Agar (LBA) (Luria-Bertani broth, Sigma-Aldrich, St. Louis, MO, USA; Agar Technical (No 2), Oxoid, Basingstoke, UK) at an optimum temperature of 27 °C and stored at length in glycerol (50% *v*/*v*) at −80 °C. Cell suspensions used throughout the experiments were prepared by collecting a loopful of AZ78 cells from the surface of 72 h old cultures and thoroughly suspending them in sterile NaCl solution (0.85% *w*/*v*). Before inoculation, a spectrophotometer (Multiskan FC, Thermo Fisher Scientific, Shanghai, China) was used to adjust the absorbance of the cell suspension to an optical density at 600 nm (A_OD600_) of 0.1, corresponding to 1 × 10^8^ cells/mL [44]. The plant pathogenic fungus *R. solani*, provided by Fondazione Edmund Mach, was routinely grown on potato dextrose agar [PDA (Potato Extract 4.0 g/L, D-Glucose 20.0 g/L, Agar 15.0 g/L, pH 5.6 ± 0.2 at 25 °C), Oxoid] at 25 °C.

### 2.2. Inhibition Assay against Rhizoctonia solani with Lysobacter capsici AZ78 Grown on Protein and Sugar-Rich Growth Media

To investigate whether the composition of growth medium may affect the volatile-mediated inhibitory activity of AZ78 against *R. solani*, we used split Petri dishes (diameter 92 mm, Sarstedt, Nümbrecht, Germany) with two adjacent but physically separated compartments. One compartment was filled with 5 mL of PDA medium, while the other with 5 mL of Nutrient Agar (NA, Oxoid) or NA supplemented with 20 g/L of D-Glucose (NAG, Sigma-Aldrich), respectively. The concentration of D-Glucose was chosen as it corresponds to the concentration of D-Glucose in the PDA medium that was previously shown to suppress the inhibitory activity of four *Lysobacter* sp. type strains against *Phytophthora infestans* [6]. NA or NAG media were inoculated with 50 μL of AZ78 cell suspension, then sealed with Parafilm (Bemis, Neenah, USA) and maintained at 25 °C. Split Petri dishes containing NA or NAG not inoculated with AZ78 cells were used as control. After 72 h incubation, NA or NAG inoculated and not inoculated with AZ78 cells were removed from the Petri dishes and a plug (5 mm in diameter) cut from the border of a seven days old *R. solani* colony was used to inoculate PDA medium. The Petri dishes were sealed and incubated at an optimum temperature of 25 °C. After 72 h, the growth inhibition of the pathogen was calculated by measuring the *R. solani* colony diameter (mm) parallel to the separation border of the two compartments and comparing it with the growth diameter of the control by applying the formula:

Mycelial growth inhibition (%) = [(diameter in control dishes − diameter in AZ78 inoculated dishes)/(diameter in control dishes)] × 100.

Five replicates (Petri dishes) were used for each treatment and the experiment was repeated twice.

### 2.3. Analysis of Volatile Organic Compounds Produced by Lysobacter capsici AZ78 on Protein-Rich and Sugar-Rich Growth Media Using Gas Chromatography-Mass Spectrometry

#### 2.3.1. Preparation of Split Petri Dishes for GC–MS Profiling

Sample preparation for GC–MS analysis was identical to Vlassi et al. [45]. Briefly, split Petri dishes containing PDA medium in one compartment and AZ78 inoculated on NA or NAG as reported above, were sealed and incubated at 27 °C for 168 h. PDA and NA or NAG, inoculated with AZ78, were then cut into pieces and transferred into sterile headspace (HS) vials (20 mL, La-Pha-Pack, Langerwehe, Germany) having sterile metal caps with 1.3 mm silicone/PTFE septa (La-Pha-Pack). The 168 h time point was chosen, as it was previously found that AZ78 growing on NA had the most abundant volatile profile [45]. HS vials that were empty and HS vials with PDA, NA or NAG medium from uninoculated Petri dishes were used as controls. A randomized block design was used and five replicates (HS vials) were analyzed for each treatment. The experiment was carried out twice with similar results. The VOC profiles of PDA and AZ78 cultures were then measured by GC–MS using the parameters indicated below.

#### 2.3.2. DHS-TD-GC-MS Analysis

GC–MS analysis was carried out on an Agilent 6890N gas chromatograph (GC, Agilent Technologies, California, USA) coupled with an Agilent 5975B quadrupole mass selective detector (MSD, Agilent). A dynamic headspace system (DHS, Gerstel), a thermal desorption unit (TDU, Gerstel) and a cooled injection system (CIS, Gerstel) were additional parts of the equipment, while the various steps of the analysis were coordinated by a multi-purpose autosampler (MPS, Gerstel, Mülheim an der Ruhr, Germany).

Analyses were carried out according to Vlassi et al. [45]. In brief, the samples to be analyzed were initially introduced to the DHS where they were first incubated (27 °C for 15 min) and subsequently VOCs were dynamically collected onto a 2 cm TENAX trap (Buchem, Apeldoorn, Netherlands) at 30 °C, while an intermediate purging step to remove water from the trap was applied. In the TDU, VOCs were thermally desorbed from the TENAX (from 30 °C to 230 °C at a rate of 60 °C/min, hold time 5 min) and the analytes were transferred to the GC–MS column following a cooled injection in the splitless mode (from −150 °C to 250 °C at a rate of 2 °C/s, hold time 6 min). The GC oven starting temperature was 35°C for 2 min, then raised to 200 °C at 5 °C/min (hold time 1 min) and increased from 200 °C to 250 °C at 20 °C/min (hold time 5 min). Chromatographic separation was carried out on a HP-5MS (5% phenyl-95% methylsiloxane) column (30 m × 0.25 mm × 0.25 μm). The carrier gas was He at a flow rate of 1 mL/min. The transfer line, ion source and quadruple temperature was set to 270 °C, 230 °C and 150 °C, respectively. The electron ionization was attained at 70 eV, at a scan range from *m*/*z* 15 to *m*/*z* 500.

The open-source software MetaboliteDetector [46], version-3.1. (http://metabolitedetector.tu-bs.de/) was used for analyzing the data generated by GC–MS as described in [45]. An in-house library of authentic standards that have been measured under the same chromatographic conditions with the samples was used for identifying the compounds, while annotation of the compounds was based on information found in NIST 14 library (National Institute of Standards and Technology, USA, http://www.nist.gov). For identification and annotation, a minimum similarity score ≥0.80 and a maximum retention index deviation (ΔRI) <5 were set as prerequisites in the software. When the search for spectral information in NIST library was not successful, literature data [47] were used for compound annotation. The RI was automatically calculated by comparison of the experimental retention time to the retention time of a series of n-alkanes (C8–C25) measured under the same sequence. Manual inspection of the detected compounds was performed and identification levels were assigned, following the criteria set by Blazenovic et al. [48]. Three confidence levels were used, more specifically confidence level (C.L.) 1, when RI and MS spectrum matched those of authentic reference standard, C.L. 2, when RI and MS spectrum matched those of literature and NIST 14 library respectively and C.L. 3, when no information on literature or NIST library could be found for the respective compound and the most likely structure is given based on comparison with literature spectra of related compounds.

### 2.4. Measurement of Ammonia in the Headspace of Bacterial Cultures

Quantofix ammonium test strips (Macherey and Nagel, Düren, Germany) were used according to Kai et al. [16] to verify the production of ammonia/amines by AZ78 cells. Briefly, a test strip was inserted into the empty compartment opposite to AZ78 cells grown on NA and/or NAG after 72 h incubation at 27 °C. The split Petri dish was sealed and incubated for 1 h at room temperature. Then, 10 μL of deionized H_2_O were added to the reaction zone on the test strips to allow ammonia/amines to be dissolved. After 30 s, 10 μL of NH_4_^+^-1 reagent [aqueous NaOH solution (32% *w*/*v*)] were added to the reaction zone. The color acquired by the reaction zone was recorded after 5 sec and compared to the Quantofix color chart indicator to determine the quantity of ammonium in the headspace.

As ammonia was suspected to be the basic volatile responsible for the volatile-mediated inhibitory activity of AZ78 [25,27], a series of aqueous solutions of ammonia were generated for the semi-quantification of this compound in the head space of the Petri dish. To this end, 5 mL of 0.2, 0.5, 1, 1.5, 2, 3, 4 or 10 mM ammonia were respectively added to one compartment of a split Petri dish and a Quantofix ammonium test strip was placed in the other. The procedure described above was followed and the color change was recorded to estimate the ammonia concentration.

Three replicates (Petri dishes) were used in each case and the complete procedure was repeated twice with similar results.

### 2.5. Targeted Gas Chromatography-Flame Ionization Analysis of Volatile Methyl Amines Produced by Lysobacter capsici AZ78

In order to investigate whether small amines were part of AZ78 volatile blend, a targeted gas chromatography-flame ionization analysis was followed. To this end, the headspace above both living cultures of AZ78 grown on NA medium in HS vials and AZ78 cultures grown on NA medium removed from Petri dishes, was measured at 72 h of incubation. The sample preparation was identical to [45]. Controls included empty HS vials and HS vials with NA medium. Synthetic trimethylamine in the form of trimethylamine hydrochloride (Sigma-Aldrich) was used as a reference standard and a series of diluted solutions (in 0.1 M HCl) of various concentrations was created. Additionally, in order to monitor any matrix effects on the detection of amines related to the NA medium, HS vials containing NA spiked with trimethylamine solutions were included in the analysis. Before measurements, both samples and trimethylamine reference standards were treated with 375 µL of 4 M NaOH/1 M KCl (pH 11) solution following the approach of Neyer et al. [49].

Samples and reference standards were measured in parallel by a head space (HP7694, Agilent)-gas chromatograph-flame ionization detector (HP5890, Agilent) (GC–FID). The analytical method used was according to Neyer et al. [49], with some modifications. Briefly, the following parameters were applied: HS oven at 70 °C, loop and transfer line at 80 °C, sample loop of 3 mL, injection at 220 °C at 100:1 split ratio; GC oven temperature starting at 40 °C (1 min) raised to 180 °C at 25 °C/min, chromatographic separation on a Rtx^®^-Volatile Amine column (60 m × 0.32 mm × 5 µm) (Restek Corporation, Pennsylvania, PA, USA), carrier gas He at 2 mL/min; flame ionization with synthetic air (400 mL/min)/H_2_ (43 mL/min) and N_2_ (9 mL/min) as make up gas; signal peak width 0.053 min and data rate at 5.000 Hz.

Three replicates (HS vials) were used and the experiment was repeated twice with similar results.

### 2.6. Inhibition Assay Using Phosphoric Acid “Trap”

Petri dishes with three compartments were used, containing either 5 mL of PDA, 5 mL of NA inoculated with AZ78 and 5 mL of 10 mM (50 μmol) aqueous phosphoric acid solution (aq. H_3_PO_4_, 85% *v*/*v*, Sigma-Aldrich), respectively. Petri dishes where either AZ78 was not inoculated or where H_2_O was added instead of H_3_PO_4_ were used as controls. The Petri dishes were then sealed and incubated at 27 °C for 72 h. Subsequently, both phosphoric acid and NA medium with AZ78 culture were removed from the Petri dish and PDA was inoculated with a 5 mm plug from a seven days old *R. solani* colony. The Petri dishes were sealed and incubated at 25 °C in the dark for 72 h. The pathogen growth inhibition was evaluated by measuring the colony diameter (mm) parallel to the central border separating the three compartments and comparing it with the growth diameter of the control as described previously (Section 2.2). Four replicates (Petri dishes) were used and the experiment was repeated twice.

### 2.7. Inhibition Assay with Ammonia in the Vapor Phase

Five mm plugs from seven days old *R. solani* colonies were inoculated on PDA (5 mL) in split Petri dishes. Subsequently, 100 μL of 500 mM or 50 mM aq. ammonia solution, corresponding respectively to 50 μmol or 5 μmol of ammonia, were added in the physically separated compartment. The concentrations applied were corresponding to the absolute concentrations measured in the air of the Petri dishes above AZ78 cultures on NA (50 μmol) or NAG (5 μmol) medium. As controls, Petri dishes with H_2_O instead of ammonia were used. After 72 h incubation at 25 °C, the diameter of *R. solani* colony parallel to the separation border of the two compartments was measured. The growth inhibition was calculated as described above (Section 2.2), and the results were compared to split Petri dishes where AZ78 was growing on NA or NAG medium. Five replicates (Petri dishes) for each treatment were used and the experiment was repeated twice.

### 2.8. pH Measurement of PDA Medium

For monitoring the pH changes, split Petri dishes containing 5 mL of PDA and 5 mL of NA or NAG were used. AZ78 cells were inoculated on NA or NAG medium as reported above, and the Petri dishes were sealed and incubated at 27 °C. Split Petri dishes containing NA or NAG not inoculated with AZ78 cells were used as control. After 72 h, a piece of pH indicator paper (VWR, Leuven, Belgium) was placed on the moist PDA and the color change was compared to the pH color scale. Five replicates (Petri dishes) were used and the experiment was repeated twice with similar results.

Additionally, the pH of PDA was monitored using a pH meter (IQ150, Spectrum Technologies, Aurora, IL, USA). Briefly, Petri dishes having two compartments were used following the same procedure and criteria reported above. After 72 h incubation at 27 °C, the electrode was placed on the surface of the PDA medium and the pH value was recorded. Prior to use, the pH meter was calibrated according to the instructions of the manufacturer using standard solutions of pH 10 and pH 7. Three replicates (Petri dishes) were used for each condition.

### 2.9. Inhibition Assay Using Alkalinized PDA

PDA was prepared according to manufacturer instructions and autoclaved. Before media solidification, a solution of 1N NaOH (Sigma-Aldrich) or aqueous ammonia solution [NH_4_OH (25% *w*/*v*), Sigma-Aldrich], were used to adjust the pH from approximately 5.6 to 8.2 or 7.6, respectively. Briefly, under the laminar flow, proper volumes of NaOH or ammonia solution were added, using a sterile Pasteur pipette, to the liquid autoclaved PDA under stirring, and the pH was adjusted to 8.2 or 7.6 by the aid of a pH meter (VWR) that had previously been washed thoroughly with EtOH (70% *v*/*v*) solution for sterilization. Subsequently, 15 mL of the alkaline media were poured in round 92 mm-Petri dishes and left under the laminar flow for drying. Prior to inoculation, the dishes were exposed to UV light and subsequently a 5 mm plug from a seven days old *R. solani* colony was used to inoculate the alkaline PDA. Regular PDA (pH 5.6 ± 0.2 at 25 °C) was used as control. After 72 h incubation at 25 °C, *R. solani* colony diameter was measured and growth inhibition was assessed as described above (Section 2.2). The pH of uninoculated PDA plates was controlled using pH paper, for an approximate indication of the alkalinity. In parallel, as positive control, inhibition was tested in split Petri dishes having AZ78 grown on NA or NAG and *R. solani* on PDA. Five replicates (Petri dishes) were used for each treatment and the experiment was repeated twice.

### 2.10. Assay Testing Multiple Factors Hypothesis

Three factors hypothesized to contribute to AZ78 volatile–mediated inhibitory activity, namely ammonia, pH value of PDA and VOC, were simultaneously tested against *R. solani*. To this end, Petri dishes with three physically separated compartments were used and PDA with pH 8.2, 100 μL of 500 mM aqueous ammonia solution and 20 μL of 250 g/L (or 5 mg) 2-ethyl-3-methoxypyrazine (Sigma-Aldrich) solution (in ACN, Sigma) were respectively added to each compartment according to the scheme reported in Table 1. 2-Ethyl-3-methoxypyrazine was used as a representative bioactive VOC of AZ78 profile, as when applied against *R. solani* inhibited the growth of the plant pathogenic fungus more efficiently among the pyrazines tested [45]. Additionally, Petri dishes with AZ78 growing initially on NA medium and subsequently *R. solani* inoculated on PDA, were used as controls of the inhibition. Five replicates (Petri dishes) were used for each treatment and the experiment was repeated twice.

### 2.11. Statistical Analysis

IBM SPSS software v. 26 (New York, NY, USA) was used for statistics. All experiments were carried out twice using three to five replicates. Prior to the statistical analysis, data of growth inhibition (%) were subjected to arcsine transformation and they were subsequently evaluated singularly for normal distribution (Shapiro-Wilk test, *p* > 0.05) and homogeneity of variance (Levene’s tests, *p* > 0.05). As both assumptions were met, data were analyzed using statistical parametric tests. Data from two independent experiments were pooled when two-way analysis of variance (ANOVA) test indicated non-significant differences (*p* > 0.05). In the case where significant differences between experiments were observed, one representative experiment was chosen for data analysis. In case of pairwise comparisons, significant differences between treatments were assessed with the Student’s *t*-test (*p* ≤ 0.05), while for multiple comparisons the Tukey’s HSD test (*p* ≤ 0.05) was used or the Games-Howell test (*p* ≤ 0.05) was used, where no homogeneity of variances was observed.

## 3. Results

### 3.1. Lysobacter Capsici AZ78 Volatiles Produced on Protein-Rich Medium Significantly Inhibit the Growth of Rhizoctonia solani

**Figure 1:**
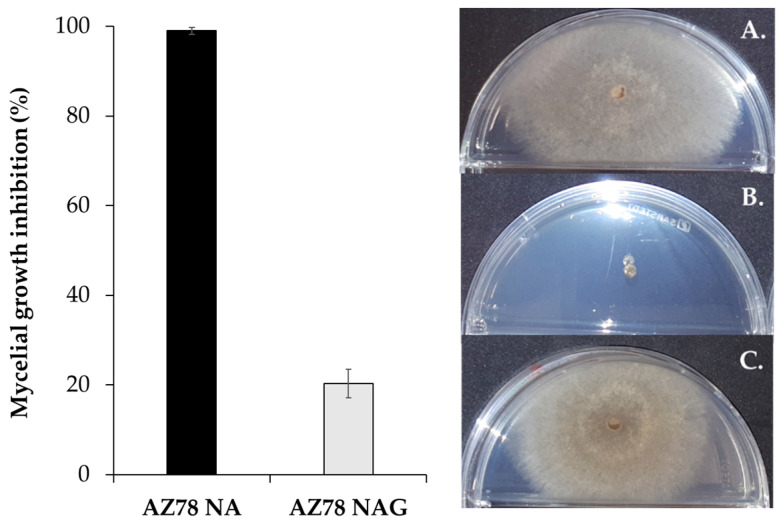
Inhibition of *Rhizoctonia solani* growth by *Lysobacter capsici* AZ78 volatiles emitted on different growth media. The side where *R. solani* had been grown is illustrated: (**A**) Control, not inoculated with AZ78; (**B**) AZ78 on Nutrient Agar and (**C**) AZ78 on Nutrient Agar amended with d-Glucose (20 g/L).

*R. solani* mycelial growth was significantly inhibited [(99.0 ± 0.8)% inhibition] by AZ78 volatiles emitted on the protein-rich NA growth medium, compared to when the glucose-rich NAG was used [(20.4 ± 3.2)% inhibition]. In the latter case, the mycelium grew to a similar extent as in the control (Figure 1).

### 3.2. Pyrazines Are Involved in the Inhibition of Rhizoctonia solani Growth by Lysobacter capsici AZ78 Grown on Protein-Rich Medium

To investigate whether the divergent *R. solani* mycelial growth was related to the different type or amount of VOCs produced by AZ78 cells grown on the protein-rich medium (NA) and sugar-rich medium (NAG), we compared the volatile profile of the bacterial cultures grown on NA and NAG by DHS-TD-GC-MS. To be confident about the metabolites directly involved in the inhibition of the plant pathogenic fungus, we analyzed the volatile profile of both AZ78 cultures and adjacent but physically separated PDA according to [45]. In total, 20 VOCs were detected in the profile of AZ78 grown in both type of media and respective PDA, 13 of which were further identified based on synthetic standards (Table S1). Sixteen out of the 20 compounds detected here, were previously identified or annotated in the VOC profile of AZ78 grown in HS vials with NA medium [45]. Pyrazines were found as the major compound class, which is also in line with our previous observations [45]. In the current study, we decided to focus on the PDA VOC profile and specifically on the compounds detected in more than half (>5/10) of the replicates measured. The compound identified in the VOC profile of half of PDA replicates from split Petri dishes where AZ78 was grown on NA was 2,5-dimethylpyrazine, while no 2,5-dimethylpyrazine was detected in any of the PDA replicates from Petri dishes with AZ78 previously grown on NAG medium. Moreover, this VOC was found to be produced at (×6) higher levels in the head space above AZ78 cultures grown on NA compared to AZ78 grown on NAG, further reinforcing its involvement in AZ78 inhibitory activity previously hypothesized [45]. On the other hand, 2-ethyl-3-methoxypyrazine was detected in similar amounts in the head space above both PDA from split Petri dishes with AZ78 grown on NA and NAG medium, while increased levels (ca. four times) of this VOC were detected above AZ78 grown on NAG (Table S1).

### 3.3. Ammonia Contributes to the Inhibition of Rhizoctonia solani Growth by Lysobacter capsici AZ78 Grown on Protein-Rich Medium

While screening the literature, we found a number of studies reporting that ammonia production by bacteria was dependent on culture media composition and specifically a positive correlation between the production of gaseous ammonia and the abundance of nitrogen content of culture media was described [17,25,27]. Thus, we speculated that ammonia may contribute to the inhibition of *R. solani* growth by AZ78. Consequently, a colorimetric NH_4_^+^ assay was used to estimate whether ammonia and/or amines were emitted in the gas phase of the Petri dish where AZ78 was growing. The measurements revealed that around 150–200 mg/L of NH_4_^+^ was produced when AZ78 had been grown on NA and around 25–50 mg/L of NH_4_^+^ when grown on NAG medium (Figure S1). Subsequently, for semi-quantitative analysis, a standard series with various concentrations of ammonia was used for calibration (Figure S2). The obtained color scale was used to determine the quantity of gaseous ammonia released by AZ78 cells grown on NA and NAG. At 72 h post inoculation (hpi), the amount of ammonia in the vapor phase of the sealed Petri dish was estimated to be approximately 50 μmol in the case of AZ78 cells grown on NA and 5 μmol in the case of NAG medium (Figure S2). Since Quantofix test (Nessler’s reagent) is not specific for ammonia but can show cross-reactivity with small amines like trimethylamine, GC-FID analysis was performed to test whether trimethylamine is present in the volatile blend of AZ78 grown on NA medium (Figure S3). The results showed that the presumable trimethylamine production, if any, would be lower than 1 μg in absolute amount which is much less than the 0.85 mg of ammonia (50 μmol) estimated to be present in the vapor phase above AZ78 grown on NA medium (Figure S3).

Subsequently, using a similar setup as Weise et al. [17], we added 50 μmol of phosphoric acid in tripartite Petri dishes with AZ78 cultures on NA medium and *R. solani* cultures on PDA medium, to trap the emitted ammonia. At 72 hpi, the inhibitory activity of AZ78 was significantly attenuated and *R. solani* growth inhibition was reduced from (96.9 ± 1.3)% (AZ78 grown on NA) to (62.2 ± 2.0)% (Table S2), confirming that basic volatiles are involved in inhibition.

To test whether the 50 μmol and 5 μmol of gaseous ammonia will inhibit the growth of the plant pathogen similar to AZ78 growing on NA or NAG respectively, corresponding amounts of aq. ammonia were added to the physically separated compartment of the split Petri dish. *R. solani* growth was inhibited by (33.0 ± 2.7)% after the application of 50 μmol ammonia and (3.0 ± 0.6)% in case of 5 μmol of ammonia (Table 2). The level of inhibition was significantly lower when compared to AZ78 growing on NA [(99.0 ± 0.8)% inhibition] and NAG medium [(20.4 ± 3.2)% inhibition], respectively.

### 3.4. Alkaline PDA Medium Impedes the Growth of Rhizoctonia solani

As a next step, the pH of the growth media used for AZ78 and *R. solani* cultivation in split Petri dishes was recorded and the pH of the physically separated PDA was shown to increase from 5.5–6 (0 hpi) to approximately 8.5–9 (72 hpi) in the case of AZ78 grown on NA and to approximately 7.5–8 (72 hpi) when grown on NAG medium (Figure S1). This initial pH indicator paper based estimation was further supported by a more accurate pH measurement with a microelectrode pH meter. PDA from split Petri dishes with AZ78 grown on NA had an average pH of 8.2 ± 0.2 (at 27 °C), while PDA from Petri dishes with AZ78 grown on NAG showed an average pH of 7.6 ± 0.1 (at 27 °C) (Table S3). PDA that had not been exposed to AZ78 emissions was used as control and was measured having a pH of 5.2 ± 0.2 (at 27 °C).

The elevation of pH was attributed to the accumulation of ammonia emitted by AZ78 in PDA and consequently the effect of alkaline PDA on *R. solani* growth was examined. When the pH of PDA was increased to 8.2 using aq. ammonia solution no growth of *R. solani* mycelium was observed (100% inhibition), while addition of ammonia in PDA to a pH of 7.6 inhibited *R. solani* mycelial growth by (35.8 ± 2.3)%. Interestingly, when NaOH was used as the alkalinizing factor, *R. solani* mycelium was inhibited by (21.3 ± 1.4)% at pH 7.6, while at pH 8.2 an inhibition of (55.4 ± 1.4)% was recorded, which was significantly less than the respective ammonia-mediated pH increase (Table 3).

### 3.5. Alkaline PDA, Ammonia and VOC May Jointly Contribute to the Inhibition of Rhizoctonia solani Growth

**Figure 2:**
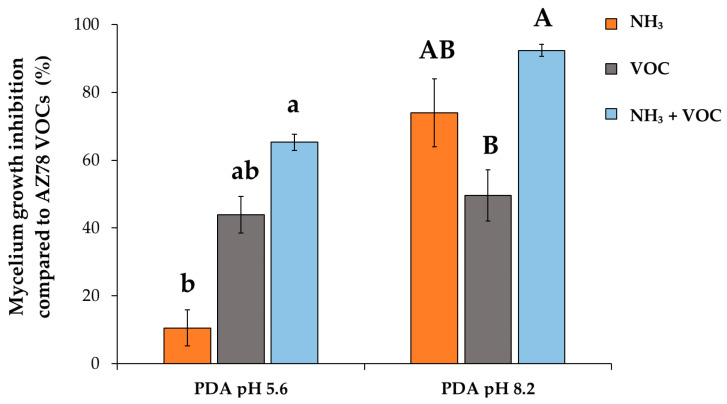
Inhibitory effect caused by multiple factors. Data presented as mean ± standard error of five replicates. Different letters indicate significant differences among treatments according to the Games-Howell test (*p* ≤ 0.05) in the case of PDA with pH 5.6 and to the Tukey’s HSD test (*p* ≤ 0.05) in the case of PDA with pH 8.2.

All three factors, namely PDA medium with pH 8.2 (after adjustment using NaOH), 50 μmol of gaseous ammonia and VOC (5 mg/Petri of 2-ethyl-3-methoxypyrazine) that appeared to contribute to the overall inhibitory activity of AZ78 against *R. solani*, were subsequently tested together. The most drastic inhibition of (92.4 ± 1.7)% of the plant pathogen growth was caused when all factors under investigation were combined (Figure 2). When ammonia was applied jointly with alkalinized PDA (pH 8.2 using NaOH), the second most notable inhibition was found at (74.0 ± 10.0)%. Except for the single VOC application, alkalinization of PDA enhanced the inhibitory activity of the treatments with gaseous ammonia and gaseous ammonia plus VOC.

## 4. Discussion

AZ78 has been formerly reported to produce various volatile pyrazines that inhibited the growth of plant pathogenic fungi and an oomycete [45]. However, when testing single pyrazines produced by AZ78, their limited inhibitory activity was pointing to other factors or compounds that may contribute as well to the bioactivity of AZ78 [45]. Moreover, a former study on the VOCs produced by four *Lysobacter* type strains indicated that the inhibitory activity against *Phytophthora infestans* was dependent on the type of growth medium used [6]. Based on these results, we aimed to investigate (1) whether the protein excess in the medium does affect the inhibitory activity of AZ78, (2) which VOCs constitute the blend emitted by AZ78 and (3) discover which compounds are differentially produced that would play a putative role in *R. solani* inhibition. Here we show that although it is obvious that VOCs contribute to the inhibitory activity of AZ78, other factors are as well playing a role in inhibition that will be discussed in detail below.

Confirming the results of Lazazzara et al. [16], the inhibitory effect of the emitted volatiles on the mycelium growth of *R. solani* was significantly enhanced when AZ78 was grown on NA medium, compared to when NA was amended with 20 g/L of d-glucose (NAG). The DHS-TD-GC-MS analysis that followed to investigate the composition of the VOC profiles revealed that pyrazines was the major group of compounds produced in both cases and that the majority of the compounds produced were similar between the two types of growth media. The compound preferentially produced by AZ78 grown on NA medium was 2,5-dimethylpyrazine but was not detected in the head space above PDA medium from Petri dishes where AZ78 was grown on NAG. These results further reinforce our previous hypothesis that pyrazines, including 2,5-dimethylpyrazine, contribute to AZ78 inhibitory activity [45]. On the other hand, 2-ethyl-3-methoxypyrazine was detected in similar amounts in the head space above PDA from split Petri dishes with AZ78 grown on NA and NAG medium, while AZ78 grown on NAG produced higher amounts of this VOC. Further, 2-ethyl-3-methoxypyrazine was shown to inhibit the mycelial growth of *R. solani* when tested as synthetic compound and was found to be more efficient—in terms of minimum dosage and maximum inhibition, than 2,5-dimethylpyrazine [45]. Thus, the differences in the type and amount of VOCs produced could not entirely explain the difference in *R. solani* inhibition observed between AZ78 grown on NA and NAG medium. Consequently, we investigated whether other volatiles are involved in the inhibitory activity of AZ78 or/and further factors may contribute to the inhibition of *R. solani* growth. In the experiments that followed, it was demonstrated that AZ78 differentially produces alkaline volatiles, according to the culture medium used and that higher amounts were released during cultivation on NA medium compared to NAG. These basic volatiles were shown to contribute to the inhibitory activity of AZ78, as demonstrated by the partial alleviation of *R. solani* inhibition following the addition of phosphoric acid in the assay. In parallel, we observed that the pH of PDA in split Petri dishes increased from pH 5.6 to pH 8.2 when AZ78 was grown in the separated compartment on NA and up to pH 7.6 when the bacterium was grown on NAG. Based on the current literature on alkaline bacterial volatile compounds we concluded that ammonia may contribute to both PDA pH increase and antifungal activity [17,25,27]. Subsequently, ammonia was confirmed to be released by AZ78 and be involved in the PDA medium alkalinization and inhibition of *R. solani*. Furthermore, it was investigated whether AZ78 also produced trimethylamine that has previously been shown to alkalinize the physically separated growth medium [50]. We found that under the tested conditions the emission of trimethylamine by AZ78, if any, would be too low to be considered in PDA alkalinization.

As *R. solani* mycelium had been reported to grow from pH 4 to pH 9 depending on the strain, while the optimum growth pH for this pathogen has been estimated to be pH 5.6 [51], experiments were conducted using NaOH solution to adjust the pH of PDA, in order to investigate to what extent the pH of the growth substrate was contributing to inhibition. At pH 7.6, *R. solani* mycelium growth was attenuated to the same degree as when AZ78 was grown on NAG in the physically separated compartment of the split Petri dish. In contrast, at pH 8.2, *R. solani* growth was inhibited significantly less when compared to the inhibition caused by AZ78 on NA. These observations lead us to speculate that alkalinization of PDA by the ammonia emissions of AZ78 is contributing only to some extent in *R. solani* growth inhibition.

Based on the semi-quantitative NH_4_^+^ assay we estimated that the gas phase inside the split Petri dishes with AZ78 grown on the protein-rich medium NA contained 50 μmol of ammonia, while only 1/10, around 5 μmol, of ammonia were found in split Petri dishes with AZ78 grown on NAG. This tenfold increase in ammonia emission can be explained by the substrate available to the bacteria for generating energy and biomass. As all heterotrophic organisms, aerobic bacteria generate energy (ATP) by oxidizing organic nutrients of their growth substrate, such as carbohydrates (e.g., glucose) and amino acids, following a process known as respiration [52]. In contrast to glucose catabolism, during utilization of amino acids for energy generation, ammonia is formed as a by-product. When amino acids are used as energy source, they are catabolized to various intermediates of the TCA cycle, like fumarate or pyruvate [52] with ammonia being generated as a result of oxidative or reductive deamination [53], hydrolysis [54] or decarboxylation [55] of the amino acids. Examples include L-aspartate and glycine that have been identified as major amino acid precursors for ammonia production in *Escherichia coli* [56] and *Streptomyces griseus* [18], respectively. The ammonia formed can then either be recycled as a nitrogen source for amino acid and nucleic acid biosynthesis or excreted from bacterial cells as a gas [52]. Thus, the excess of nitrogen-containing molecules in NA growth medium probably drives AZ78 to use amino acids as an energy source and this favors the formation of ammonia. Similar findings have been reported for *Pseudomonas* sp. [27].

In the rhizosphere of plants, AZ78 could potentially encounter conditions with an excess of amino acids or proteins. For bacteria, root exudates of crop plants for example are a major source of both amino acids and sugars, along with organic acids, phenolic compounds and minerals [57,58,59]. Interestingly, increased amounts of amino acids and decreased production of sugars have been observed in *Arabidopsis thaliana* root exudates, following foliar inoculation with *Pseudomonas syringae* [60]. Furthermore, most soilborne plant pathogenic fungi, including *R. solani*, are necrotrophs [61] that survive by killing and feeding on cells of host plants, commonly of the roots. Following plant cell lysis, amino acids would be released to the rhizosphere [62]. Additionally, the attack strategies of AZ78 against plant pathogenic oomycetes involve the use of proteolytic and other enzymes for cell wall degradation and eventually cell lysis [42]. In all the above scenarios, a local enrichment of amino acids/peptides could occur in the rhizosphere, which might favor the formation of ammonia by AZ78 cells, resulting in high ammonia concentrations in situ that could directly antagonize the nearby plant pathogenic microorganisms. Interestingly, an enhanced production of ammonia in soil amended with the amino acid glycine, has already been observed in vitro for *Streptomyces griseus* [18].

Ammonia, as a small neutral molecule, can passively diffuse through cell membranes and lipid bilayers [63], influencing fungal or oomycete cells in various ways. High ammonia influx into fungal cells, will potentially result in increased pH of cytoplasm and organelles [64]. In filamentous fungi, special plasma membrane P-ATPase transport proteins regulate the intracellular pH and maintain a proper ion balance across the cytoplasmic membrane [65], as an increased intracellular pH can negatively influence vital cellular processes, including the enzymatic transformations, the configuration of proteins, ATP synthesis and nutrient import [66]. Moreover, fungal cells possess specific membrane located ammonium/ammonia transporters (MEPs), as in the case of *Saccharomyces cerevisiae*, *F. fujikuroi* and *Aspergillus nidulans* [67,68,69] that regulate the ammonia diffusion in the cells [63]. Due to these equilibrating mechanisms, in the case of a high ammonia influx, a respective high ammonium/ammonia efflux will probably follow, resulting in a high consumption rate of chemical energy, with negative effects on cell functions, as it has been demonstrated in plants [70]. Additionally, evidence of direct disruption of the fungal cell membrane integrity by high gaseous ammonia concentrations has been reported that resulted in mycelium electrolyte leakage and respiration inhibition [71]. Hence, ammonia can have detrimental effects on *R. solani* cells and although, as discussed above, probably certain regulatory mechanisms can counteract the negative consequences, it seems that there is a certain regulatory capacity limit (i.e., concentration threshold) above which the fungal cells are no longer capable of homeostasis.

This hypothesis is reflected in our findings, as little or no growth was observed when AZ78 was grown on NA medium and the PDA pH increased to 8.2 presumably by the release of ammonia. The seemingly small difference between pH 7.6 and pH 8.2 is accompanied by a tenfold increase in gaseous ammonia concentration inside the Petri dish and consequently higher accumulation in the PDA medium, suggesting a higher ammonia influx into *R. solani* cells that further supports the various scenarios leading to growth inhibition. Thus, the significant differences in mycelial growth observed between cultivation on alkalinized PDA with pH 7.6 and PDA with pH 8.2, can largely be attributed to the detrimental effects of high ammonia concentration. Furthermore, the significant difference between the inhibitory effect of alkalinized PDA (pH 8.2) by the addition of NaOH and of ammonia are further supporting the hypothesis that both direct exposure to ammonia as well as elevated pH contribute to the higher growth inhibition of *R. solani* mycelium with AZ78 growing on NA medium. Our findings are in accordance with previous studies where the germination of conidia of *F. graminearum* and *Penicillium griseofulvum* was inhibited by ammonium bicarbonate at high pH values, as a result of the release of high concentrations of ammonia, while the increased extracellular pH was not considered as the sole reason for inhibition, as conidia viability was not remarkably affected by PDA with buffered respective pH values [72]. Similarly, the germination of *Verticillium dahliae* microsclerotia was attenuated with increased concentrations of ammonium and pH values and likewise, less germination was observed by increasing concentrations of ammonia in the agar medium [73].

The structure of soil and of the rhizosphere that can in some cases favor the accumulation of volatile molecules in soil pores [74], and, additionally, the continuous production and release of volatiles by bacteria like AZ78, support the possibility of ammonia to accumulate to concentrations acutely toxic to plant pathogenic microorganisms. On the other hand, several factors in the soil environment could potentially reduce the amounts of ammonia accumulated, including ammonia decomposition by ammonia-oxidizing bacteria or incorporation into humic acids [75]. In this case, the inhibitory effect could be impeded, as it was demonstrated by the moderate inhibitory activity observed following single application of 50 μmol aq. ammonia in the split Petri dish and additionally by the decrease of AZ78 inhibitory activity when phosphoric acid was present in the Petri dish. Moreover, it should be considered that the soil pH and soil buffering capacity, also play a crucial role in the equilibrium between gaseous ammonia and the ammonium cation. Alkaline soils supplied with ammonia-releasing nitrogenous amendments showed an enhanced suppressiveness [76]. Similarly, our experiment with the combination of 50 μmol of aq. ammonia and PDA with pH 8.2 adjusted by NaOH, resulted in an inhibition of *R. solani* growth significantly higher than the ammonia application combined with regular PDA with pH 5.6. Adding further to the complexity of the conditions found in the rhizosphere environment, soil pH is constantly influenced by root exudates [77]. Hence, it is probably more realistic to state that in soil, the complementary effect of ammonia and substrate pH will contribute to AZ78 inhibitory activity. Ammonia emissions, VOC emissions and substrate pH were shown here for the first time to interact and drastically inhibit the growth of *R. solani* in vitro. Thus, although in the closed set-up of the Petri dish the accumulation in PDA of ammonia emitted by AZ78 grown on NA medium is presumably the major inhibitory factor, our findings suggest that under real-world conditions it is likely that the volatile-mediated inhibition caused by AZ78 in protein-rich substrates can be achieved by the interaction of multiple factors, including inorganic ammonia, VOCs and substrate pH.

## 5. Conclusions

In conclusion, when grown on a protein-rich medium in vitro, AZ78 produces ammonia high enough to alkalinize the PDA medium of the adjacent compartment and sufficiently suppress the growth of *R. solani.* The plant pathogenic fungus is secondarily inhibited by alkaline pH and the combination of ammonia and alkaline pH proved to be crucial for inhibition. The synergistic effects of ammonia, alkaline pH and VOC were demonstrated that under certain conditions, probably found in the rhizosphere, could lead to the inhibition of plant pathogens from a distance. Our findings point to the involvement of ammonia and substrate pH in addition to VOCs, in the volatile-mediated inhibitory activity of rhizobacteria and intensify the complexity of bacterial-fungal interactions in the rhizosphere niche, where nutrient availability and pH can be constantly variable. Based on the results found in this research study, future experiments shall be carried out using substrates and conditions closer to the natural soil environment to further develop and test this hypothesis.

## Figures and Tables

**Table 1:** Conditions chosen for testing the involvement of multiple factors interaction for *Rhizoctonia solani* growth inhibition.

Factor	Condition							
	1	2	3	4	5	6	7	8
NH_3_^a^	+	+	-	-	+	+	-	-
VOC ^b^	+	-	+	-	+	-	+	-
pH	5.6 ^c^	5.6	5.6	5.6	8.2 ^d^	8.2	8.2	8.2

^a^ aq. solution of NH_3_ was used at 50 μmol; ^b^ 5 mg of 2-ethyl-3-methoxypyrazine per Petri dish were used as a representative volatile organic compound (VOC); ^c^ PDA pH as set by manufacturer; ^d^ PDA pH was raised to 8.2 by NaOH solution.

**Table 2:** Involvement of gaseous ammonia produced by *Lysobacter capsici* AZ78 in *Rhizoctonia solani* growth inhibition.

Treatment	Mycelial Growth Inhibition (%)
AZ78 on NA medium	(99.0 ± 0.8)
AZ78 on NAG medium	(20.4 ± 3.2) ^b^
50 μmol NH_3_	(33.0 ± 2.7) ^a^
5 μmol NH_3_	(3.0 ± 0.6) ^c^

Inhibition of *R. solani* growth was monitored at 72 hpi; data presented as mean ± standard error of ten replicates pooled from two independent experiments; different letters indicate significant differences among treatments according to the Tukey’s HSD test (*p* ≤ 0.05).

**Table 3:** Involvement of elevated PDA pH in *Rhizoctonia solani* growth inhibition by *Lysobacter capsici* AZ78.

Treatment	Mycelial Growth Inhibition (%)
AZ78 on NA medium	(99.0 ± 0.8) *
PDA pH 8.2 by NaOH	(55.4 ± 1.4)
PDA pH 8.2 by NH_3_	100 *
AZ78 on NAG medium	(20.4 ± 3.2) ^b^
PDA pH 7.6 by NaOH	(21.3 ± 1.4) ^b^
PDA pH 7.6 by NH_3_	(35.8 ± 2.3) ^a^

Inhibition of *R. solani* growth was monitored at 72 hpi. Data presented as mean ± standard error of ten replicates pooled from two independent experiments; different letters indicate significant differences according to the Tukey’s HSD test (*p* ≤ 0.05). * As no growth of *R. solani* mycelium was observed in most of split Petri dishes after AZ78 had been grown on NA as well as in all Petri dishes in which the pH value of PDA had been adjusted to 8.2 by NH_3_, these two treatments were excluded from the statistical analysis.

## Supplementary Materials

The following are available online at https://www.mdpi.com/2076-2607/8/11/1761/s1, Table S1: DHS-TD-GC-MS analysis of split Petri dishes with *Lysobacter capsici* AZ78 cultures and PDA, Table S2: Involvement of basic volatiles produced by *Lysobacter capsici* AZ78 in the inhibition of *Rhizoctonia solani* growth, Table S3: PDA alkalinization as a result of *Lysobacter capsici* AZ78 basic volatile emissions, Figure S1: Production of basic volatiles by *Lysobacter capsici* AZ78 at 72 h incubation, Figure S2: Standard series of ammonia concentrations, Figure S3: Parallel chromatographic analysis (HS-GC-FID) of *Lysobacter capsici* AZ78 samples grown on NA medium (168 h) and synthetic trimethylamine.
