# Supplementary material for: Volatile-Mediated Inhibitory Activity of Rhizobacteria as a Result of Multiple Factors Interaction: The Case of Lysobacter capsici AZ78
Source: Microorganisms. 2020 Nov 9;8(11):1761. doi: 10.3390/microorganisms8111761 (PMC7695267; doi:10.3390/microorganisms8111761)
Supplement: Supplementary file 1 [file microorganisms-08-01761-s001.pdf]

Table S1 DHS-TD-GC-MS analysis of split Petri dishes with *Lysobacter capsici* AZ78 cultures and PDA.

						Split Petri dish				Abundance ratio	
						AZ78 [a]		PDA [b]		AZ78 [c]	PDA [d]
Nr	CAS	Metabolite*	C.L.[1]	RI[2]	Sim[3]	NA	NAG	NA	NAG	(NA/NAG)	
1	123-51-3	3-Methyl-1-butanol	1	753	0.90	–	5/10	–	1/10	–	–
2	109-97-7	Pyrrole	1	768	0.84	1/10	3/10	2/10	1/10	0.11	0.07
3	868-57-5	Methyl 2-methylbutanoate	1	779	0.86	–	4/10	–	–	–	–
4	123-32-0	2,5-Dimethylpyrazine	1	912	0.80	10/10	10/10	5/10	–	6.31	–
5	63012-97-5	2-Methyl-3-(methylthio)-furan	1	947	0.98	–	4/10	–	–	–	–
6	928-68-7	6-Methyl-2-heptanone	2	954	0.96	3/10	–	1/10	–	–	–
7	2847-30-5	2-Methyl-3-methoxypyrazine	1	971	0.87	3/10	4/10	1/10	4/10	0.13	0.37
8	2882-21-5	6-Methyl-2-methoxypyrazine	2	983	0.96	2/10	–	1/10	–	–	–
9	108-75-8	2,4,6-Trimethylpyridine	1	995	0.84	2/10	1/10	–	–	9.22	–
10	104-76-7	2-Ethyl-1-hexanol	1	1028	0.88	4/10	9/10	–	–	0.65	–
11	816-19-3	Methyl 2-ethylhexanoate	2	1042	0.97	–	6/10	–	–	–	–
12	25680-58-4	2-Ethyl-3-methoxypyrazine	1	1051	0.86	7/10	10/10	6/10	6/10	0.40	1.27
13	19846-22-1	3-Methoxy-2,5-dimethylpyrazine	1	1057	0.94	–	8/10	2/10	1/10	–	2.09
14	25773-40-4	2-Isopropyl-3-methoxypyrazine	1	1094	0.84	2/10	3/10	3/10	2/10	0.05	0.77
15	–	Isopropyl-methoxy-methylpyrazine derivative	3	1157	0.96	1/10	3/10	2/10	–	0.57	–
16	24168-70-5	2-*Sec*-butyl-3-methoxypyrazine	1	1172	0.88	–	3/10	1/10	1/10	–	0.50
17	24683-00-9	2-Isobutyl-3-methoxypyrazine	1	1179	0.98	–	3/10	1/10	–	–	–
18	–	Ethyl-dimethyl-methoxypyrazine derivative	3	1209	0.99	–	2/10	1/10		–	–
19	–	3-*Sec*-butyl-2-methoxy-5(6)-methylpyrazine	2	1229	0.96	3/10	9/10	2/10	2/10	0.29	2.72
20	–	Isopropyl-dimethyl-methoxypyrazine derivative	3	1237	1.00	–	4/10	–	–	–	–

* Compounds related to control (growth medium and ambient air) or considered to be artifacts of the analytical procedure, were excluded from the list; [a,b] Number of sample replicates the VOC was detected in, to the total replicate number of [a] AZ78 cultures grown on NA or NAG medium and of [b] PDA from split Petri dishes with AZ78 grown in a separate compartment on NA or NAG medium respectively; [c,d] Abundance ratio as peak area of compounds in [a]AZ78 cultures on NA to NAG medium and [b] PDA from split Petri dishes with AZ78 cultures grown on NA to PDA from split Petri dishes with AZ78 cultures grown on NAG medium; [1] Confidence levels assigned according to Blazenovic et al. [48]; [2]Average retention index of ten replicates pooled from two independent experiments; [3]Average similarity score of ten replicates pooled from two independent experiments.

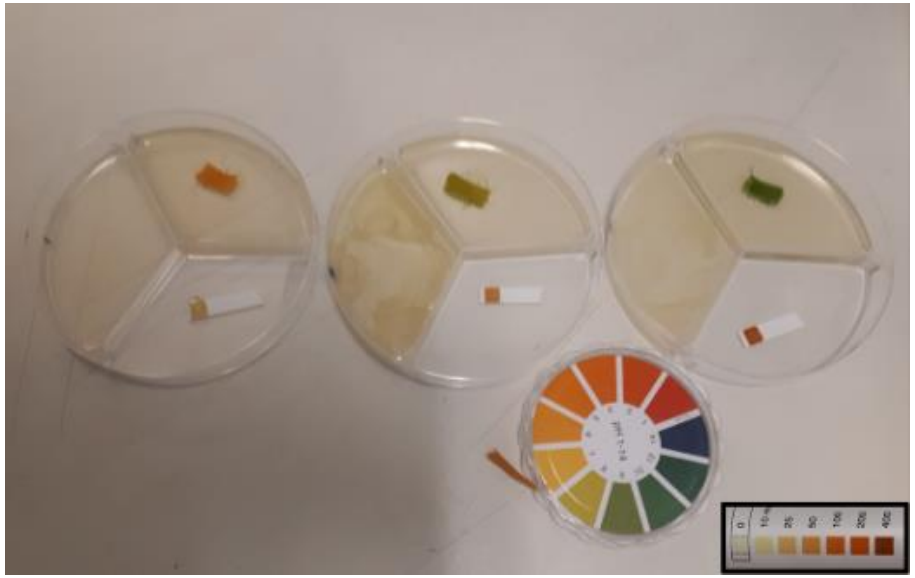

Figure S1 Production of basic volatiles by *Lysobacter capsici* AZ78 at 72 h incubation. pH paper coloration indicating PDA alkalinization and separated Quantofix NH_4^+ results (colored tip of white stick with reaction site, color scale at the bottom right). Control (no AZ78 inoculation) (left), AZ78 grown on NAG (middle) and AZ78 grown on NA medium (right).

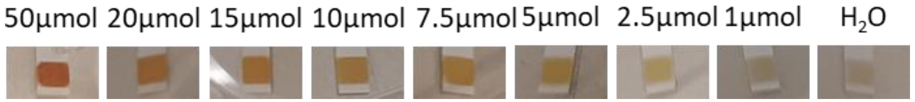

Figure S2 Standard series of ammonia concentrations.

Table S2 Involvement of basic volatiles produced by *Lysobacter capsici* AZ78 in the inhibition of *Rhizoctonia solani* growth.

Treatment	Mycelial growth inhibition (%)
AZ78 on NA medium	(96.9 ± 1.3)
Addition of 50 µmol H_3PO_4	(62.2 ± 2.0)*

Inhibition of *R. solani* growth was monitored at 72 hpi; Data presented as mean ± standard error of five replicates; Asterisk indicates significant differences between treatments according to the Student's t-test ($p \leq 0.05$).

Table S3 PDA alkalinization as a result of *Lysobacter capsici* AZ78 basic volatile emissions.

AZ78 growth medium	PDA pH
Nutrient Agar	8.2 ± 0.2
Nutrient Agar plus D-Glucose (20 $g.L^{-1}$)	7.6 ± 0.1

The pH was monitored at 72 h of AZ78 incubation; Data presented as mean ± standard deviation of three replicates.

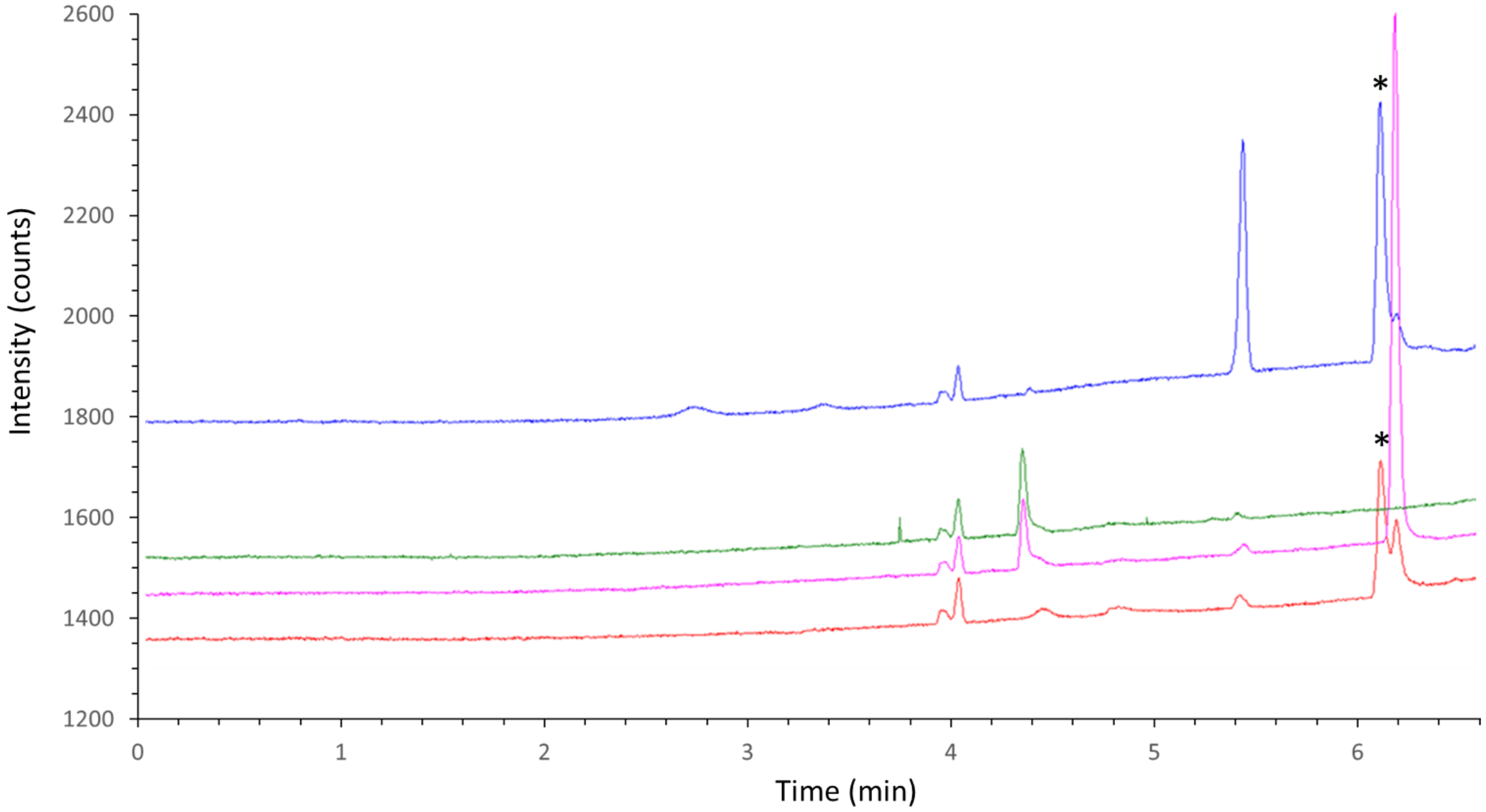

Figure S3 Parallel chromatographic analysis (HS-GC-FID) of *Lysobacter capsici* AZ78 samples grown on NA medium (168 h) and synthetic trimethylamine. Chromatograms overlay of AZ78 grown on NA in HS vials (green line), AZ78 grown on NA in Petri dishes and subsequently placed in HS vials for analysis (purple line), trimethylamine (*) spiked in NA at 8 µg (blue line) and trimethylamine (*) at 1 µg (red line).
